# Selective Subnormal IgG1 in 54 Adult Index Patients with Frequent or Severe Bacterial Respiratory Tract Infections

**DOI:** 10.1155/2016/1405950

**Published:** 2016-03-31

**Authors:** James C. Barton, Luigi F. Bertoli, J. Clayborn Barton, Ronald T. Acton

**Affiliations:** ^1^Department of Medicine, Brookwood Medical Center, Birmingham, AL 35209, USA; ^2^Southern Iron Disorders Center, Birmingham, AL 35209, USA; ^3^Department of Medicine, University of Alabama at Birmingham, Birmingham, AL 35209, USA; ^4^Brookwood Biomedical, Birmingham, AL 35209, USA; ^5^Department of Microbiology, University of Alabama at Birmingham, Birmingham, AL 35294, USA

## Abstract

We characterized 54 adult index patients with reports of frequent or severe bacterial respiratory tract infections at diagnosis of selective subnormal IgG1. Mean age was 50 ± 13 (SD) y; 87.0% were women. Associated disorders included the following: autoimmune conditions 50.0%; hypothyroidism 24.1%; atopy 38.9%; and other allergy 31.5%. In 35.5%, proportions of protective* S. pneumoniae *serotype-specific IgG levels did not increase after polyvalent pneumococcal polysaccharide vaccination (PPPV). Blood lymphocyte subset levels were within reference limits in most patients. Regressions on IgG1 and IgG3 revealed no significant association with age, sex, autoimmune conditions, hypothyroidism, atopy, other allergy, corticosteroid therapy, or lymphocyte subsets. Regression on IgG2 revealed significant associations with PPPV response (negative) and CD19+ lymphocytes (positive). Regression on IgG4 revealed significant positive associations with episodic corticosteroid use and IgA. Regression on IgA revealed positive associations with IgG2 and IgG4. Regression on IgM revealed negative associations with CD56+/CD16+ lymphocytes. Regressions on categories of infection revealed a negative association of urinary tract infections and IgG1. HLA-A^⁎^03, HLA-B^⁎^55 and HLA-A^⁎^24, HLA-B^⁎^35 haplotype frequencies were greater in 38 patients than 751 controls. We conclude that nonprotective* S. pneumoniae* IgG levels and atopy contribute to increased susceptibility to respiratory tract infections in patients with selective subnormal IgG1.

## 1. Introduction

Immunoglobulin (Ig) G subclass deficiency (IgGSD) is a clinically and genetically heterogeneous disorder characterized by frequent or severe bacterial infections of the upper and lower respiratory tract [1–3]. Selective subnormal IgG1 levels have been reported in adults and children, many of whom presented with recurrent respiratory tract infections [4–7]. Some adults with selective subnormal IgG1 also have autoimmune conditions [6], atopy [6], or suboptimal responses to polyvalent pneumococcal polysaccharide vaccination (PPPV) [7]. IgG1 comprises ~53% of serum total IgG [8]. Anti-infection properties of IgG1 include its capacity to bind soluble and membrane protein antigens with relative specificity, its moderate reactivity to polysaccharide antigens, its high-affinity binding to Fc receptors on macrophages, and its ability to activate complement [9].

We sought to extend characterization of adults with selective subnormal IgG1. Thus, we retrospectively compiled clinical and laboratory features in 54 consecutive white adult index patients referred to a single practice because they had frequent or severe bacterial respiratory tract infections. We describe age at diagnosis, sex, specialties of referring physicians, autoimmune conditions, prevalence of atopy and other allergy manifestations, responses to PPPV, corticosteroid therapy, levels of serum Ig isotypes, blood lymphocyte subset levels, and human leukocyte antigen- (HLA-) A and human leukocyte antigen- (HLA-) B types. Our results are discussed in the context of previous reports of clinical and genetic features of other patients with similar clinical and laboratory phenotypes and putative alleles that could modulate serum IgG1 levels.

## 2. Methods

### 2.1. Patient Selection

The performance of this work was approved by the Institutional Review Board of Brookwood Medical Center. All patients reported herein were referred to a hematology/medical oncology practice for further evaluation and management because they had the following: (a) frequent or severe bacterial respiratory tract infections uncontrolled by antibiotic therapy and other management; (b) subnormal total serum IgG or subnormal IgG1.

We defined IgGSD as serum levels of one or more IgG subclasses (IgG1–IgG3) at least 2 standard deviations (SD) below the mean(s) for age in the presence of normal serum IgG, with or without subnormal serum IgA [3]. From such patients, we included only those who had subnormal serum IgG1 levels in the absence of subnormal IgG2, IgG3, IgG4, IgA, or IgM. Many also had nonprotective serotype-specific serum IgG levels for or impaired responses to* Streptococcus pneumoniae* polysaccharide antigens.

We included all white adults from central Alabama (≥18 years of age) referred to as outpatients in the interval January 2000–April 2015 who had frequent or severe bacterial infections, typically of the upper and lower respiratory tract, and who were diagnosed to have subnormal serum levels of one or more Ig isotypes classified as IgGSD [3, 10, 11]. We designated the first persons in respective families diagnosed to have IgGSD as index patients. We recommended that all patients accept vaccination with Pneumovax (Pneumovax23^®^ (PPPV); Merck, Sharpe & Dohme, Whitehouse Station, NJ) as an in vivo means of evaluating IgG response to polysaccharide antigens [3, 10, 12]. We did not routinely test for responses to protein antigens, for example, tetanus toxoid.

We observed these categories of infection reports: sinusitis; bronchitis; pneumonia; otitis media; pharyngitis; tonsillitis; bronchiectasis; laryngitis; skin; oral cavity/teeth; genitourinary tract; gastrointestinal tract; and central nervous system. We neither evaluated nor treated patients for infections before they were referred for immunology assessments reported herein.

Autoimmune conditions, atopy, and other allergy manifestations were diagnosed and characterized by referring physicians, our queries at initial consultation, and medication reviews. Herein, we defined atopy as allergic asthma, allergic rhinitis, or allergic eczema. Other allergy manifestations included urticaria, angioedema, or anaphylaxis in association with treatment with certain medications, ingestion of specific foods, or exposure to specific nonfood environmental allergens. Some patients reported recurrent urticaria or other allergy manifestations unassociated with exposure to known allergens. We tabulated patient reports of first-degree family members who had frequent or severe respiratory tract infections or autoimmune conditions.

We defined corticosteroid therapy in the 54 index patients using three dichotomous variables: daily oral steroids prescribed for management of autoimmune conditions; intermittent oral or parenteral steroids, usually prescribed to relieve manifestations of infection; and topical or inhaled corticosteroids, typically used intermittently for diverse indications.

### 2.2. Patient Exclusions

We excluded white patients with the following: (a) hypogammaglobulinemia attributed to B-cell neoplasms, organ transplantation, immunosuppressive therapy, anticancer chemotherapy, or increased Ig loss; (b) monoclonal gammopathy; (c) human immunodeficiency virus infection; (d) inability to complete pretreatment evaluation.

### 2.3. Laboratory Methods

Testing was performed before IgG replacement therapy was initiated. Serum Ig levels were measured using standard clinical methods at a single reference laboratory (Laboratory Corporation of America, Burlington, NC). We defined mean ± 2 SD as the normal or reference range for all Ig measurements, consistent with other investigators [8, 11, 13, 14]. Reference ranges for Igs are displayed in Table 1 footnotes. Subnormal Ig levels were defined as those below the corresponding lower reference limit. Subnormal serum IgG1 levels were documented twice in all patients at times they did not have acute infections. We elected use values of the second IgG subclass panel for the present analyses.

Pre- and post-PPPV* S. pneumoniae *serotype-specific IgG antibodies were measured by clinical laboratories (Laboratory Corporation of America, Burlington, NC, and ViraCor-IBT, Lee's Summit, MO). Diluents for patient samples tested for* S. pneumoniae* serotype-specific IgG contained C-polysaccharide and polysaccharide type 22. The median interval between pre- and post-PPPV* S. pneumoniae* IgG test panels was 43 days (range 19–203 d). In patients who received PPPV, none received IgG therapy before post-PPPV testing was reported. Test panels included measurements of antibodies specific for 6, 7, or 14 serotypes (Supplementary Table 1 in the Supplementary Material available online at http://dx.doi.org/10.1155/2016/1405950). We defined serotype-specific IgG levels as either protective (≥1.3 mg/L) or nonprotective (<1.3 mg/L) [15].

Blood levels of lymphocyte subsets were measured using flow cytometry. Reference ranges are displayed in Table 2 footnotes. Subnormal levels and elevated levels were defined as those below and above the corresponding lower reference limits, respectively.

HLA-A and HLA-B alleles were detected using low-resolution DNA-based typing (polymerase chain reaction/sequence-specific oligonucleotide probe) in index patients and family members to define haplotypes as described in detail elsewhere [11].

Data from 1,321 apparently normal, unrelated Caucasian adult subjects from Alabama who had undergone HLA-A and HLA-B phenotype analysis as part of paternity testing were used to estimate frequency of allele positivity [16]. HLA-A and HLA-B haplotypes were determined in 751 unrelated Caucasian subjects from Alabama who had undergone testing to establish paternity as described in detail elsewhere [16]. Preliminary analyses revealed that frequencies of the major HLA-A and HLA-B haplotypes observed in the present control subjects were similar to those in Caucasians from a large national bone marrow donor program [17].

### 2.4. Statistics

The final analytic data set consisted of observations on 54 index patients with selective subnormal IgG1 (and of HLA-A and HLA-B haplotypes in 34 index patients). Responses to PPPV were defined as follows: (1) any increase in the number of protective antibody levels after PPPV (dichotomous variable; 31 index patients); (2) percentage increments of the numbers of protective antibody levels in the post-PPPV panel compared to those in the pre-PPPV panel (continuous variable).

Analyses were performed with Excel 2000^®^ (Microsoft Corp., Redmond, WA) and GB-Stat^®^ (v. 10.0, 2003, Dynamic Microsystems, Inc., Silver Spring, MD). D'Agostino's test was used as a measure of normality. Descriptive data are displayed as enumerations, percentages, mean ± 1 standard deviation (SD), median (range), or mean (95% confidence intervals (CI)). Age at diagnosis data were normally distributed and were compared using Student's *t*-test (two-tailed). Because some measures of serum Ig isotypes and blood lymphocyte subsets were not normally distributed, we compared these data using the Mann-Whitney *U* test. Proportions were compared using Pearson's *χ* ^2^ test or Fisher's exact test, as appropriate. Linear correlations were performed using Pearson's technique. We computed relative risks (RR) (95% CI) or odds ratios (OR) (95% CI) for some observations. We performed backward stepwise regression on serum levels of IgG1, IgG2, IgG3, IgG4, IgA, and IgM using these independent variables, as appropriate: age; sex; autoimmune conditions; hypothyroidism; atopy; other allergy manifestations; IgG1; IgG2; IgG3; IgG4; IgA; IgM; CD19+, CD3+, CD4+, CD8+, and CD56+ lymphocytes; daily oral steroids; episodic oral or parenteral steroids; topical or inhaled corticosteroids; and response to PPPV. We defined values of *p* < 0.05 to be significant. Bonferroni corrections were applied to control type I error rate at 0.05 for separate comparisons of continuous and dichotomous data, as appropriate.

## 3. Results

### 3.1. General Characteristics of 54 Index Patients

There were 7 men (13.0%) and 47 women (87.0%). The mean age of all index patients was 50 ± 13 (SD) y. The mean age of men was 54 ± 17 y and that of women was 49 ± 13 (*p* = 0.5000). Primary care, otolaryngology, rheumatology, and pulmonology specialists referred 94.4% of all patients.

Typical patient reports of bacterial respiratory tract infection substantiated by records of referring physicians documented four or more episodes yearly requiring outpatient antibiotic therapy (and other management), one or more serious or life-threatening respiratory tract infections yearly, or a combination of these manifestations. Many patients and referring physicians reported inadequate response of infections to antibiotic therapy. Upper and lower respiratory tract infections interpreted as bacterial were reported by these proportions of index patients: sinusitis 92.6%; bronchitis 77.8%; pneumonia 53.7%; otitis media 35.2%; pharyngitis 27.8%; tonsillitis 29.6%; bronchiectasis 7.4%; and laryngitis 1.9%. Two or more sites of respiratory tract infection were reported by 90.7% of index patients.

Some patients also reported that they had frequent or severe bacterial, viral, or* Candida* infections at other sites, including skin (46.3%; cellulitis, abscesses due to methicillin-resistant and sensitive* Staphylococcus aureus*, and herpes zoster), oral cavity/teeth (37.0%; intraoral herpes, candidiasis, and excessive gingival and apical infections), genitourinary tract (27.8%; urinary tract infection, candidiasis, and labial abscess), gastrointestinal tract (3.7%;* Helicobacter pylori* gastritis,* Clostridium difficile* colitis, and colonic diverticulitis), and central nervous system (1.9%; bacterial meningitis after head trauma). The mean ages (y) ± SD of patients at diagnosis of selective subnormal IgG1 with these categories of infection were as follows: skin, 40 ± 13 y; oral cavity/teeth, 51 ± 8 y; genitourinary tract, 54 ± 12 y; gastrointestinal tract, 44 ± 22 y; and central nervous system, 64 y. Patients who reported* Candida* infections (oral or vulvovaginal) were 50 ± 12 y old at diagnosis of selective subnormal IgG1. We (and patients) did not have ages at which most of their infections occurred.

At diagnosis of subnormal IgG1, 5.6% of index patients reported taking daily oral steroids, 35.2% reported taking episodic oral or parenteral steroids, and 11.1% reported taking topical or inhaled corticosteroids.

### 3.2. Autoimmune Conditions

One-half of the present patients (27/54; 50.0%) had one or more autoimmune conditions. The proportions of men and women who had one or more autoimmune conditions did not differ significantly (data not shown). The respective prevalence of 15 categories of autoimmune disorders in men and women did not differ significantly (data not shown). Sjögren's syndrome, systemic lupus erythematosus, Hashimoto's thyroiditis, interstitial cystitis, mixed connective tissue disorder, Raynaud's phenomenon, and rheumatoid arthritis accounted for 76.0% of the autoimmune conditions. Hypothyroidism not otherwise specified was reported in 11 women (23.4%) and no men (*p* = 0.1820).

### 3.3. Atopy and Other Allergy Manifestations

Twenty-one patients (38.9%) had atopy, including allergic asthma (*n* = 17), allergic rhinitis (*n* = 2), and allergic eczema (*n* = 2). No patient had more than one atopic condition. Seventeen patients (31.5%) had other allergy manifestations, including urticaria, angioedema, and anaphylaxis. Eleven patients had these manifestations in association with treatment with certain medications, 6 patients had these manifestations with exposure to specific nonfood environmental allergens, and 4 patients reported having recurrent urticaria or other allergy manifestations unassociated with exposure to known allergens. None reported allergy manifestations associated with foods. Some patients had more than one allergy. Eight patients (14.8%) had both atopy and other allergy manifestations. Twenty-four patients (44.4%) had had neither atopy nor other allergy manifestations.

### 3.4. Family Histories

Reports of frequent or severe respiratory tract infections and autoimmune conditions in first-degree family members were obtained from 23 patients (42.6%) and 11 patients (20.4%), respectively. The respective proportions of these family reports did not differ significantly between men and women (data not shown).

### 3.5. *Streptococcus pneumoniae* Serotype-Specific IgG Antibodies

Observations were available in 50 of 54 patients (92.6%). All pre-PPPV IgG antibodies in test panels were “protective” in four of 50 patients (8.0%). Panels of both pre- and post-PPPV* S. pneumoniae *serotype-specific IgG antibodies were available in 31 patients (64.8%). The proportion of protective IgG levels did not increase after PPPV in 11 of these 31 patients (35.5%). The median positive change in the percentages of protective IgG levels after PPPV in 31 patients was 14.3% (mean 22.4%). Thus, most patients achieved little or no increase in protective levels of serotype-specific IgG antibodies after PPPV.

### 3.6. Serum IgG, IgG Subclasses, IgA, and IgM

The Pearson correlation coefficient of IgG by the sum of IgG1–IgG4 levels was 0.8689 (*p* < 0.0001). The median total IgG level was 709 mg/dL (range 386, 1114). Total IgG was subnormal in 25 patients (46.3%). No patient had elevated IgG2 or IgG4. IgG3 and IgA were elevated in one patient each. Five patients (9.3%) had elevated IgM (Table 1).

### 3.7. Univariable Comparisons of Ig Levels

Median levels of IgG subclasses, IgA, and IgM did not differ significantly between men and women or between patients with and without autoimmune conditions, atopy, other allergy manifestations, episodic oral or parenteral steroids, or topical or inhaled corticosteroids (data not shown). Median IgA was lower in three patients who reported that they took daily oral steroids (95 mg/dL (80, 100)) than in 51 patients who did not take daily oral steroids (162 mg/dL (84, 489)) (*p* = 0.0192). Median IgG2 was lower in 20 patients who had responses to PPPV (188 mg/dL (125, 559)) than in 34 patients who did not respond to PPPV (314 mg/dL (131, 724)) (*p* = 0.0012).

### 3.8. Blood Lymphocytes

Levels of CD19+, CD3+/CD4+, CD3+/CD8+, and CD56+/CD16+ lymphocytes were not subnormal in any patient. Median levels of these lymphocyte subsets did not differ significantly between men and women (Table 2). The proportions of men and women who had CD19+ and CD3+/CD4+ lymphocyte levels outside the respective reference limits were low (Table 2).

### 3.9. Regressions on Ig Levels at Diagnosis

Regression on IgG1 revealed no significant associations with age, sex, autoimmune conditions, hypothyroidism, atopy, other allergy manifestations, IgG2, IgG3, IgG4, IgA, IgM, lymphocyte subsets, steroid therapy, or response to PPPV. Regression on IgG2 revealed two associations: response to PPPV (negative association; *p* = 0.0064); CD19+ blood lymphocytes (positive association; *p* = 0.0267). This regression explained 14.8% of the variance of IgG2 (ANOVA *p* = 0.0041). Regression on IgG3 revealed no significant associations. Regression on IgG4 revealed two positive associations: episodic use of corticosteroids (*p* = 0.0226); IgA (*p* = 0.0034). This regression explained 13.2% of the variance of IgG4 (ANOVA *p* = 0.0069). Regression on IgA revealed two positive associations: IgG2 (*p* = 0.0384); IgG4 (*p* = 0.0254). This regression explained 13.2% of the variance of IgA (ANOVA *p* = 0.0069). Regression on IgM was negatively associated with CD56+/CD16+ lymphocytes (*p* = 0.0352). This regression explained 8.2% of the variance of IgM (ANOVA *p* = 0.0352).

### 3.10. Relationships of Ig Levels to Types of Infections

We performed logistic regressions on each category of infection reports using IgG subclasses, IgA, and IgM levels as independent variables. IgG1 was negatively associated with reports of urinary tract infections (*p* = 0.0207; OR 0.99 (0.97; 1.00)). There were negative associations of IgM with bronchitis (*p* = 0.0196; OR 0.99 (0.97, 1.00)) and skin infections (*p* = 0.0402; OR 0.99 (0.98, 1.0)). We detected no significant association of IgG subclasses, IgA, and IgM with other categories of infections.

### 3.11. HLA Types and Haplotypes in Patients and Control Subjects

HLA-A and HLA-B typing was available in all 54 index patients. Some patients were homozygous for HLA-A types: A^*∗*^01 (5.6%); A^*∗*^02 (14.8%); and A^*∗*^03 (1.9%). Positivity for HLA-A types in patients and control subjects did not differ significantly (Supplementary Table 2).

Some patients were homozygous for HLA-B types: B^*∗*^08 (5.6%); B^*∗*^15 (1.9%); B^*∗*^18 (1.9%); and B^*∗*^44 (7.4%). Positivity for B^*∗*^15 and B^*∗*^40 was greater in patients than in controls (Table 3). These two HLA-B types were observed in 31.5% of index patients. RR associated with these two respective types were as follows: 12.9 (6.3, 26.3) (*p* < 0.0001); 4.9 (2.3, 10.5) (*p* < 0.0001). Positivity for B^*∗*^62 was lower in index patients (Table 3), although the RR was not reduced (0.07 (0.005, 1.1) (*p* = 0.0600)).

HLA-A and HLA-B haplotypes were available for 38 of the 54 index patients (64.8%). One patient was homozygous for A^*∗*^01, B^*∗*^08 and another patient was homozygous for A^*∗*^02, B^*∗*^15. Three patients were homozygous for A^*∗*^02, B^*∗*^44. After Bonferroni correction, the haplotypes A^*∗*^03, B^*∗*^55, and A^*∗*^24, B^*∗*^35 were more prevalent in index patients than in 751 controls (Table 4).

## 4. Discussion

General characteristics of the present 54 index patients are similar to those of 57 French adults with selective subnormal IgG1 and frequent respiratory tract infections [6]. Selective subnormal IgG1 also occurred in 24 Dutch patients with recurrent sinopulmonary infections [7]. In contrast, infection risk is not increased in all adults with selective subnormal IgG1 [6]. In the present patients, increased susceptibility to bacterial infection is probably related to multiple factors, including subnormal IgG1 levels, decreased reactivity to PPPV, and high prevalence of allergic asthma and allergic rhinitis. Specific antibody deficiency that extends to other IgG subclasses may also contribute to infection susceptibility.

Encapsulated bacteria, primarily* S. pneumoniae* and* Haemophilus influenzae* type b (Hib), account for a large proportion of serious infections in persons with primary immune deficiency [6, 18–22], including those with selective subnormal IgG1 [6]. Antibodies produced to the polysaccharide capsule are type-specific and protect against infections [23–27]. In one study, IgG1 in seven commercial intravenous IgG preparations contained nearly as much anti-pneumococcal antibody as IgG2 on a per-milligram-of-IgG-subclass basis [28]. Anti-pneumococcal polysaccharide antibodies of the IgG1 and IgG3 subclasses occur in persons who have IgA deficiency [29] or heavy chain constant region deletions of the *γ*2 gene [30, 31]. IgG1 anti-Hib polysaccharide from adults killed Hib more effectively in vitro than IgG2 [32]. In children, immunization with Hib polysaccharide vaccine elicited both IgG1 and IgG2 responses, with a slight predominance of IgG1 [33]. Taken together, these observations are consistent with anti-infection properties of IgG1 [2, 34].

Most of the present patients achieved little or no increase in protective levels of serotype-specific IgG antibodies after PPPV. In 51 other persons with recurrent sinopulmonary infections and mild IgG1 subclass deficiency IgG1 antipolysaccharide responses were also impaired [7]. Responses to PPPV also vary among normal adults [35]. In normal adults immunized with PPPV,* S. pneumoniae* IgG antibodies are predominantly those of the IgG2 subclass [36, 37]. Increases in serotype-specific IgG1 and IgG3 antibodies occur after immunization [37]. In the present patients, PPPV responses were negatively associated with IgG2 levels. In 24 Dutch patients with selective subnormal IgG1, IgG2 antipolysaccharide responses were significantly lower in patients with histories of pneumonia [7]. Risks for infections due to particular serotypes of* S. pneumoniae* may be increased in patients who cannot synthesize specific protective antibodies [7, 38, 39], consistent with the present observations.

One-half of the present patients had one or more autoimmune conditions. Autoimmune conditions were also common in another cohort of persons with selective subnormal IgG1 [6] and in other case series of IgGSD [39–41]. The aggregate prevalence of 29 autoimmune conditions in the United States general population is estimated to be 7.6–9.4% [42]. This suggests that the prevalence of autoimmune conditions is greater in adults with selective subnormal IgG1 than in the general population.

Atopy occurred in more than one-third of the present patients. Asthma occurred in 31% of the present patients and in 20% of 119 children and adults with selective subnormal IgG1 [6]. Atopy and asthma were prominent manifestations in 57 other adults with selective subnormal IgG1 [6]. Allergic asthma increases risk of lower respiratory tract infections in subjects not selected for subnormal immunoglobulin levels [43–45]. In one study, viral or bacterial infections were detected in 70% of inpatients with exacerbation of asthma [46]. Likewise, allergic rhinitis increases the risk of sinusitis [47].

There was a preponderance of women (87%) in the present 54 patients. The proportion of women among 57 French adults with selective subnormal IgG1 was also high (61%) [6]. Our observations substantiate but do not explain the preponderance of women among adults who have selective subnormal IgG1 and frequent or severe respiratory tract infections.

Hypothyroidism occurred in 24% of index patients. Prevalence estimates of hypothyroidism in large population studies in which screening of whites for thyroid disorders was performed were 1.3% [48] to 4.6% (0.3% clinical, 4.3% subclinical) [49]. Thus, the prevalence of hypothyroidism may be greater in patients with than in those without diagnoses of selective subnormal IgG1.

Episodic corticosteroid therapy reported by the present index patients was positively associated with IgG4. In children with aplastic anemia or autoimmune conditions treated with prednisolone for more than 2 months, mean serum IgG1 was as high as that of normal controls [50]. Other observations indicate that corticosteroid therapy sometimes reduces total IgG and serum IgG1 levels depending on steroid dose and schedule, underlying conditions (if any), and site of IgG synthesis [51–53].

Elevated IgM was observed in 10% of the present patients, although none had serum protein electrophoresis and immunofixation studies interpreted as monoclonal IgM. Nonclonal elevated serum IgM levels occur in many conditions, including rheumatoid arthritis [54–58]. This could explain elevated IgM in some of the present patients. The clinical features and laboratory immunophenotypes of hyper-IgM disorders types 1–5 [59–63] are inconsistent with those of the present patients.

Quantitative deficits of blood lymphocytes that would explain increased susceptibility to respiratory tract bacterial infections were detected in few of the present patients. Subnormal CD3+/CD8+ lymphocytes or subnormal CD56+/CD16+ blood lymphocytes occurred in only 2% and 6% of patients, respectively.

Positivity for HLA-B^*∗*^15 and HLA-B^*∗*^40 was associated with increased OR for selective subnormal IgG1. The HLA haplotypes A^*∗*^03, B^*∗*^55 and A^*∗*^24, B^*∗*^35 were more prevalent in index patients than in 751 controls. Putative alleles that modulate serum IgG1 levels may be linked to certain HLA-A and HLA-B loci on chromosome 6p.

*IGHG1* (chromosome 14q32.33) encodes the IgG1 heavy chain. The main allelic forms for IgG1 are G1m (z,a), G1m (f), and G1m (f,a) [9, 64, 65]. The G1m (f) allele is only found in Caucasians, whereas the G1m (f,a) allele is common in Asians; other variants have also been described [9, 66, 67]. Plasma IgG concentrations are correlated with Gm allotypes [68, 69] and IgG allotypes can influence clinical manifestations of immunity [9, 70].

Lacombe et al. observed selective subnormal IgG1, with or without other Ig deficits, in first-degree family members of some index patients with selective IgG1 deficiency [6]. Vertical transmission of IgGSD linked to HLA haplotypes has been demonstrated in other kinships [11, 71, 72]. These reports are consistent with our observation that 43% of the present patients reported that they had first-degree family members who had frequent or severe respiratory tract infections. Twenty-percent of the present patients reported that they had first-degree relatives with autoimmune conditions. This is consistent with family histories of autoimmune conditions reported by index patients with selective subnormal IgG3 [41].

Uncertainties in the present work include identity of the microbes that caused many respiratory tract infections reported by the present patients and their physicians before diagnosis of selective subnormal IgG1. The prevalence of atopy varies widely according to age, gender, and geographic region [73, 74]. Accordingly, it is unknown whether the present patients had higher rates of asthma, allergic rhinitis, and eczema than other Alabama adults. Flow cytometry analysis of subsets of CD19+, CD3+/CD4+ and CD3+/CD8+, and CD56+/CD16+ blood lymphocytes and functional studies of lymphocytes may have demonstrated additional abnormalities. HLA types and haplotypes vary across geographic regions and across race/ethnicity groups [75, 76]. Thus, HLA studies may yield dissimilar results in different cohorts of patients with selective subnormal IgG1. Performing extended HLA haplotyping and G1m allotyping was beyond the scope of the present work.

## 5. Conclusions

The present 54 index patients with selective subnormal IgG1 were characterized by preponderance of women, prominence of autoimmune conditions, hypothyroidism, atopy, other allergy manifestations, and decreased response to PPPV. Subnormal IgG1, nonprotective* S. pneumoniae* IgG levels, and a high prevalence of allergic asthma and allergic rhinitis contribute to increased susceptibility to bacterial respiratory tract infections in the present patients. Increased risk for selective subnormal IgG1 may be linked to HLA-A and HLA-B loci and G1m allotypes.

## Supplementary Material

Table 1: Test panels included measurements of antibodies specific for 6, 7, or 14 serotypes.
Table 2: Positivity for HLA-A types in patients and control subjects did not differ significantly.

## Figures and Tables

**Table 1:** Serum immunoglobulins in 54 adults with selective subnormal IgG1^1^.

Ig isotype	Men (*n* = 7)	Women (*n* = 47)	Value of *p*
Median IgG, g/L	6.71 (5.51, 8.84)	7.13 (3.86, 11.14)	0.2212
Elevated IgG, % (*n*)	0	0	~1

Median IgG1, g/L	3.37 (2.93, 4.10)	3.78 (0.95, 4.19)	0.2794
Elevated IgG1, % (*n*)	0	0	~1

Median IgG2, g/L	2.41 (1.61, 4.01)	2.28 (1.25, 7.24)	0.7968
Elevated IgG2, % (*n*)	0	0	~1

Median IgG3, g/L	0.50 (0.43, 1.76)	0.59 (0.41, 1.18)	0.3342
Elevated IgG3, % (*n*)	14.3 (1)	0	0.1296

Median IgG4, g/L	0.12 (0.01, 0.46)	0.09 (0.02, 0.64)	0.6993
Elevated IgG4, % (*n*)	14.3 (1)	0	0.1296

Median IgA, mg/L	181 (90, 256)	154 (80, 489)	0.8368
Elevated IgA, % (*n*)	0	2.1 (1)	0.8704

Median IgM, mg/L	147 (44, 408)	100 (40, 277)	0.6246
Elevated IgM, % (*n*)	14.3 (1)	8.5 (4)	0.5150

^1^By definition, all patients had subnormal IgG1 and no patient had subnormal IgG2, IgG3, IgG4, IgA, or IgM.

Reference ranges are as follows: IgG 7.0–16.0 g/L (700–1600 mg/dL); IgG1 4.2–12.9 g/L (422–1292 mg/dL); IgG2 1.2–7.5 g/L (117–747 mg/dL); IgG3 0.4–1.3 g/L (41–129 mg/dL); IgG4 0–2.9 g/L (1–291 mg/dL); IgA 700–4000 mg/L (70–400 mg/dL); and IgM 400–2300 mg/L (40–230 mg/dL). Subnormal Ig levels were defined as those below the corresponding lower reference limit. Serum Ig levels are expressed as median (range). Elevated serum Ig levels were defined as those greater than the upper reference limit. Comparisons were made with Mann-Whitney *U* test, Pearson's *χ* ^2^ test, or Fisher's exact test, as appropriate.

**Table 2:** Blood lymphocyte subsets in 54 adults with selective subnormal IgG1^1^.

Lymphocyte subset	Men (*n* = 7)	Women (*n* = 47)	Value of *p* ^2^
Median CD19+ cells/*µ*L	160 (76, 432)	228 (64, 576)	0.5281
Subnormal CD19+ cells, % (*n*)	(0)	(0)	~1
Elevated CD19+ cells, % (*n*)	(0)	(0)	~1

Median CD3+/CD4+ cells/*µ*L	1295 (371, 2058)	934 (442, 2984)	0.5976
Subnormal CD3+/CD4+ cells, % (*n*)	(0)	(0)	~1
Elevated CD3+/CD4+ cells, % (*n*)	14.3 (1)	10.6 (5)	0.5843

Median CD3+/CD8+ cells/*µ*L	641 (237, 1082)	430 (48, 1046)	0.1643
Subnormal CD3+/CD8+ cells, % (*n*)	(0)	2.1 (1)	0.8704
Elevated CD3+/CD8+ cells, % (*n*)	14.3 (1)	6.4 (3)	0.4360

Median CD56+/CD16+ cells/*µ*L	98 (17, 376)	146 (14, 401)	0.4173
Subnormal CD56+/CD16+ cells, % (*n*)	14.3 (1)	4.3 (2)	0.3463
Elevated CD56+/CD16+ cells, % (*n*)	(0)	(0)	~1

^1^Blood levels of lymphocyte subsets were measured using flow cytometry. Reference ranges (mean ± 2 SD) are as follows: CD19+ 12–645 cells/*μ*L; CD3+/CD4+ 359–1,519 cells/*μ*L; CD3+/CD8+ 109–897 cells/*μ*L; and CD56+/CD16+ 24–406 cells/*μ*L. Levels are expressed as median (range). Subnormal levels were defined as those below the corresponding lower reference limits. Elevated levels were defined as those greater than the upper reference limits. Comparisons were made with Mann-Whitney *U* test, Pearson's *χ* ^2^ test, or Fisher's exact test, as appropriate.

^2^These are nominal values of *p*. Bonferroni correction for 12 comparisons yielded a revised *p* for significance of <0.0042.

**Table 3:** HLA-B positivity in Alabama adults^1^.

Type	Selective subnormal IgG3 (*n* = 54)	Population control (*n*)	Value of *p* ^2^
B^*∗*^07	16.7 (9)	0.2259 (1321)	0.3082
B^*∗*^08	29.6 (16)	0.2487 (1315)	0.4286
B^*∗*^13	1.9 (1)	0.0343 (1314)	0.4493
B^*∗*^14	0	0.0639 (1314)	0.0304
B^*∗*^15	18.5 (10)	0.0144 (1320)	<0.0001
B^*∗*^18	0	0.0775 (1251)	0.0160
B^*∗*^27	0	0.0895 (1318)	0.0070
B^*∗*^35	22.2 (12)	0.1406 (1309)	0.0938
B^*∗*^37	3.7 (2)	0.0240 (1167)	0.3865
B^*∗*^38	3.7 (2)	0.0193 (1198)	0.2937
B^*∗*^39	0	0.0225 (1198)	0.3002
B^*∗*^40	13.0 (7)	0.0265 (1321)	<0.0001
B^*∗*^41	0	0.0119 (1089)	0.5339
B^*∗*^44	24.1 (13)	0.2866 (1263)	0.4644
B^*∗*^45	3.7 (2)	0.0177 (1241)	0.2643
B^*∗*^47	0	0.0039 (513)	0.8184
B^*∗*^49	1.9 (1)	0.0171 (1230)	0.6145
B^*∗*^50	0	0.0140 (1212)	0.4743
B^*∗*^51	9.3 (5)	0.0696 (1250)	0.5183
B^*∗*^52	1.9 (1)	0.0180 (1164)	0.6345
B^*∗*^53	0	0.0077 (1163)	0.6637
B^*∗*^55	5.6 (3)	0.0259 (1042)	0.1803
B^*∗*^56	0	0.0097 (928)	0.5998
B^*∗*^57	9.3 (5)	0.0385 (1014)	0.4127
B^*∗*^58	0	0.0256 (1014)	0.2553
B^*∗*^60	0	0.1206 (1086)	0.0126
B^*∗*^62	0	0.1285 (1175)	0.0007

^1^HLA, human leukocyte antigen. Results are displayed as % (*n*). Comparisons were made with Pearson's chi-square test or Fischer's exact test, as appropriate.

^2^These are nominal values of *p*. Bonferroni correction for 27 comparisons yielded a revised *p* for significance of <0.0019.

**Table 4:** HLA haplotype frequencies in Alabama adults^1^.

HLA-A	HLA-B	Selective subnormal IgG1 index patients (76 chromosomes)	Controls (*n* chromosomes)	Value of *p* ^2^
01	08	0.1842 (14)	0.0925 (1502)	0.0118
	37	0.0132 (1)	0.0050 (1210)	0.3478
	52	0.0132 (1)	0 (1502)	0.0488
	57	0.0132 (1)	0.0066 (1210)	0.4231
02	07	0.0263 (2)	0.0413 (1502)	0.3944
	15	0.0789 (6)	0.0306 (1502)	0.0356
	18	0.0132 (1)	0.0083 (1210)	0.4897
	27	0.0395 (3)	0.0083 (1210)	0.0369
	35	0.0132 (1)	0.0083 (1210)	0.0488
	40	0.0395 (3)	0.0073 (1502)	0.0266
	44	0.0921 (7)	0.0633 (1502)	0.3182
	45	0.0132 (1)	0 (1502)	0.0488
	51	0.0132 (1)	0.0127 (1502)	0.6297
	57	0.0263 (2)	0.0047 (1502)	0.0662
03	07	0.0526 (4)	0.0546 (1502)	0.6000
	14	0.0132 (1)	0.0113 (1502)	0.5908
	44	0.0132 (1)	0.0127 (1502)	0.6297
	55	0.0395 (3)	0 (1502)	0.0001
11	14	0.0132 (1)	0 (1502)	0.0488
	18	0.0132 (1)	0.0020 (1502)	0.1793
	35	0.0526 (4)	0.0140 (1502)	0.0291
	38	0.0132 (1)	0 (1502)	0.0488
	51	0.0263 (2)	0.0033 (1210)	0.0443
23	18	0.0132 (1)	0 (1502)	0.0488
	40	0.0132 (1)	0 (1502)	0.0488
24	7	0.0132 (1)	0.0050 (1210)	0.3478
	35	0.0132 (1)	0.0060 (1502)	0.0007
	44	0.0132 (1)	0.0083 (1210)	0.4897
26	35	0.0132 (1)	0 (1502)	0.0488
29	44	0.0395 (3)	0.0233 (1502)	0.2755
31	40	0.0132 (1)	0.0033 (1210)	0.2629
32	8	0.0132 (1)	0 (1502)	0.0488
	40	0.0132 (1)	0 (1502)	0.0488
33	44	0.0132 (1)	0 (1502)	0.0488

^1^HLA, human leukocyte antigen. These data represent observations in 38 unrelated white index patients with selective subnormal IgG1 for whom haplotyping observations were available (=76 chromosomes) and other white adults who underwent haplotype analysis for paternity testing. All subjects were residents of Alabama. All haplotypes could be detected with both DNA-based and serologic methods with the exception of haplotypes containing B^*∗*^70 and B^*∗*^72 that were not detected by serologic methods. Results are displayed as % (*n*). Comparisons were made with Fischer's exact test or Pearson's chi-square test, as appropriate.

^2^These are nominal values of *p*. Bonferroni correction for 34 comparisons yielded a revised *p* for significance of <0.0015.

## Abbreviations

ANOVA: Analysis of variance
CI: Confidence interval
HLA: Human leukocyte antigen
Ig: Immunoglobulin
IgGSD: IgG subclass deficiency
OR: Odds ratio
PPPV: Polyvalent pneumococcal polysaccharide vaccination
RR: Relative risk
SD: Standard deviation.
