# Supplementary material for: Selective Subnormal IgG1 in 54 Adult Index Patients with Frequent or Severe Bacterial Respiratory Tract Infections
Source: J Immunol Res. 2016 Mar 31;2016:1405950. doi: 10.1155/2016/1405950 (PMC4830719; doi:10.1155/2016/1405950)
Supplement: Supplementary file 1 — Table 1: Test panels included measurements of antibodies specific for 6, 7, or 14 serotypes. Table 2: Positivity for HLA-A types in patients and control subjects did not differ significantly. [file 1405950.f1.pdf]

Supplementary Table 1. *Streptococcus pneumoniae* serotype-specific IgG test panels[1]

No. of serotypes	Serotypes
6	1, 3, 14, 19, 23, 51
7	4, 14, 19, 23, 26, 56, 68
14	1, 3, 4, 8, 9, 12, 14, 19, 23, 26, 51, 56, 57, 68

[1]Serotype panels varied according to the year of diagnosis, physician choice, and insurance requirements,

Supplementary Table 2. HLA-A positivity in Alabama adults[1]

Type	Selective subnormal IgG3 (n = 54)	Population controls (n)	Value of p[2]
A*01	37.0 (20)	0.3397 (1,319)	0.6407
A*02	50.0 (27)	0.5201 (1,310)	0.7748
A*03	18.5 (10)	0.2739 (1,318)	0.1503
A*11	18.5 (10)	0.1136 (1,320)	0.1081
A*23	3.7 (2)	0.0375 (1,254)	0.6705
A*24	9.3 (5)	0.1304 (1,265)	0.4164
A*25	1.9 (1)	0.0297 (1,281)	0.5257
A*26	3.7 (2)	0.0634 (1,278)	0.3325
A*28	0	0.0773 (1,320)	0.0142
A*29	9.3 (5)	0.0620 (1,290)	0.3658
A*30	1.9 (1)	0.0405 (1,308)	0.3577
A*31	5.6 (3)	0.0350 (1,258)	0.3046
A*32	7.4 (4)	0.0536 (1,232)	0.3381
A*33	3.7 (2)	0.0176 (1,248)	0.2623
A*34	0	0.0044 (1,146)	0.7939

[1]HLA, human leukocyte antigen. Results are displayed as % (n). Comparisons were made with Pearson's chi-square test or Fischer's exact test, as appropriate.

[2]These are nominal values of p. Bonferroni correction for 15 comparisons yielded a revised p for significance of <0.0033.
